# Predicting post-operative vault and optimal implantable collamer lens size using machine learning based on various ophthalmic device combinations

**DOI:** 10.1186/s12938-023-01123-w

**Published:** 2023-06-15

**Authors:** Xi Chen, Yiming Ye, Huan Yao, Chang Liu, Anqi He, Xiangtao Hou, Keming Zhao, Zedu Cui, Yan Li, Jin Qiu, Pei Chen, Ying Yang, Jing Zhuang, Keming Yu

**Affiliations:** State Key Laboratory of Ophthalmology, Zhongshan Ophthalmic Center, Guangdong Provincial Key Laboratory of Ophthalmology and Visual Science, Guangdong Provincial Clinical Research Center for Ocular Diseases, Sun Yat-Sen University, Guangzhou, People’s Republic of China

**Keywords:** Implantable collamer lens, Machine learning, Multi-device data, Vault prediction, Size selection

## Abstract

**Background:**

Implantable Collamer Lens (ICL) surgery has been proven to be a safe, effective, and predictable method for correcting myopia and myopic astigmatism. However, predicting the vault and ideal ICL size remains technically challenging. Despite the growing use of artificial intelligence (AI) in ophthalmology, no AI studies have provided available choices of different instruments and combinations for further vault and size predictions. This study aimed to fill this gap and predict post-operative vault and appropriate ICL size utilizing the comparison of numerous AI algorithms, stacking ensemble learning, and data from various ophthalmic devices and combinations.

**Results:**

This retrospective and cross-sectional study included 1941 eyes of 1941 patients from Zhongshan Ophthalmic Center. For both vault prediction and ICL size selection, the combination containing Pentacam, Sirius, and UBM demonstrated the best results in test sets [*R*^2^ = 0.499 (95% CI 0.470–0.528), mean absolute error = 130.655 (95% CI 128.949–132.111), accuracy = 0.895 (95% CI 0.883–0.907), AUC = 0.928 (95% CI 0.916–0.941)]. Sulcus-to-sulcus (STS), a parameter from UBM, ranked among the top five significant contributors to both post-operative vault and optimal ICL size prediction, consistently outperforming white-to-white (WTW). Moreover, dual-device combinations or single-device parameters could also effectively predict vault and ideal ICL size, and excellent ICL selection prediction was achievable using only UBM parameters.

**Conclusions:**

Strategies based on multiple machine learning algorithms for different ophthalmic devices and combinations are applicable for vault predicting and ICL sizing, potentially improving the safety of the ICL implantation. Moreover, our findings emphasize the crucial role of UBM in the perioperative period of ICL surgery, as it provides key STS measurements that outperformed WTW measurements in predicting post-operative vault and optimal ICL size, highlighting its potential to enhance ICL implantation safety and accuracy.

**Supplementary Information:**

The online version contains supplementary material available at 10.1186/s12938-023-01123-w.

## Introduction

Implantable Collamer Lens (ICL, STAAR Surgical) surgery has been proven safe, effective, and predictable in correcting myopia and myopic astigmatism [1, 2]. The proper ICL size selection provides a safe post-operative vault, the distance between the center of the posterior ICL surface and the center of the anterior crystalline lens surface. The current consensus is that the ideal ICL vault ranges from 250 µm to 750 μm [3]. Higher and lower post-operative vaults are risk factors for angle-closure glaucoma and anterior subcapsular cataracts [4]. To reduce the risk of ICL post-operative complications, several researchers have proposed statistical regression methods to improve the accuracy of vault prediction for ICL sizing [5–8], including the NK [9] and KS [10] formulas. However, all these predicting formulas are based on relatively few variables and do not reflect practical information about the space, where the lens is fixed [5]. Moreover, most formulas rely on linear regression, which is used for finding the linear relationship between the target and one or more predictors. Clinical experience suggests that predicting post-operative vault is non-linear and fairly complicated [7, 8, 11]. Therefore, the pre-operative biometric variables may not exhibit a simple linear correlation with the post-operative vault, and linear regression has limitations in explaining the relationships between measurements. Consequently, predicting the vault and ideal ICL size remains technically challenging.

Artificial intelligence (AI) has recently enabled more accurate inference and higher efficiency based on extensive training data for medical applications [12, 13]. Machine learning (ML), a subset of AI, is used to predict unknown information using algorithms that learn the intrinsic statistical patterns and structures of data. Supervised ML algorithms, a sub-category of ML methods, could consider multiple features and minimize human variation for clinical decision-making [14]. Several studies have suggested that supervised ML methods have great potential for post-operative vault prediction and ideal ICL size selection [15–18]. For example, Kamiya et al*.* and Kang et al*.* demonstrated that ML of pre-operative biometric data obtained by anterior segment optical coherence tomography (AS-OCT) might be beneficial for predicting the actual ICL vault and subsequently selecting the proper ICL size [15, 16]. Yang et al*.* found that ML is applicable for vault prediction and ICL sizing based on Pentacam HR metrics [17]. However, these previous studies relied heavily on one specific piece of equipment and ignored the fact that different hospitals and clinics have different examination instruments and capabilities. Some ophthalmology departments do not have Pentacam or have more than one Scheimpflug device, such as Sirius, which combines a Scheimpflug camera with a Placido disk. Whether ML of pre-operative Sirius biometric data would benefit ICL-related prediction remains obscure. Moreover, with the widespread application of high-frequency ultrasound bio-microscopy (UBM), directly measuring the ciliary sulcus-to-sulcus (STS) distance has become possible. Previous studies have demonstrated that the STS-based method predicts post-operative vault significantly better than the traditional white-to-white (WTW)-based formula [7, 19]. Therefore, the predictive potential of using UBM parameters alone or in combination with other modal data with the aid of AI still requires further evaluation. Current AI studies have not yet explored the potential choices of different instruments and combinations for vault and ICL size predictions.

In light of the background mentioned above, we developed multiple predictive models that incorporate clinical parameters obtained from various ophthalmic devices (UBM, Pentacam, Sirius) and combinations to predict the post-operative ICL vault and select the optimal ICL size. The current study is academically and clinically meaningful, and it might be a critical step toward aiding various hospitals in minimizing the risk of post-operative complications of ICL implantation.

## Results

### Demographic characteristics in vault prediction cohort and optimal ICL size prediction cohort.

Pre-operative patient demographics are summarized in Table 1. The data set included 1941 eyes of 1941 patients (1446 females and 495 males) for post-operative vault prediction. Moreover, we further screened 1287 eyes within the ideal post-operative vault range (vault ranges from 250 µm to 750 μm) for optimal ICL size modeling. We randomly divided these eyes, allocating 80% to the training and cross-validation set and the remaining 20% to the test set.

**Table 1:** Demographic characteristics of patients regarding vault and ICL size predictions

Characteristic	Post-operative vault prediction	Optimal size prediction
Eyes, *n*	1941	1287
Patients, *n*	1941	1287
Sex		
Male, *n*	495 (25.50%)	325 (25.25%)
Female, *n*	1446 (74.50%)	962 (74.75%)
Age, years	26.32 ± 5.03	26.43 ± 5.00
Achieved ICL size		
12.1 mm (%)	372 (19.17%)	304 (23.62%)
12.6 mm (%)	957 (49.30%)	682 (52.99%)
13.2 mm (%)	525 (27.05%)	267 (20.75%)
13.7 mm (%)	60 (3.09%)	20 (1.55%)
Post-operative ICL vault, μm	624.58 ± 245.69	525.28 ± 126.31

### Machine learning algorithms based on various ophthalmic device combinations could accurately predict post-operative vault.

Most models achieved excellent performance of vault predictions, except for the groups that did not depend on UBM, Pentacam, and Sirius (Table 2). The CatBoost Regressor and Extra Trees Regressor demonstrated favorable performance in predicting vault for most device combinations within our study. The combination of UBM, Pentacam, and Sirius outperformed single-device or dual-device combinations in our regression models [validation set: *R*^2^ = 0.504 (95% CI 0.480–0.527), MAE = 129.893 (95% CI 127.758–132.046); test set: *R*^2^ = 0.499 (95% CI 0.470–0.528), MAE = 130.655 (95% CI 128.949–132.111)], and the absence of these three devices resulted in poor predictions [validation set: *R*^2^ = 0.208 (95% CI 0.175–0.242), MAE = 175.310 (95% CI 169.631–181.099); test set: *R*^2^ = 0.210 (95% CI 0.181–0.240), MAE = 172.132 (95% CI 167.290–176.934)]. These results indirectly showed that the three devices had a substantial contribution to vault prediction. When only parameters from a single device were allowed for prediction, the stacking strategy based on Sirius achieved outstanding performance.

**Table 2:** Performance of the regression models for post-operative vault prediction

Device (s)	Top 3 algorithms	Validation set		Test set	
		R^2^ (95% CI)	MAE (95% CI)	R^2^ (95% CI)	MAE (95% CI)
UBM	et	0.398 (0.380 to 0.416)	145.056 (142.161 to 147.961)	0.400 (0.382 to 0.418)	144.856 (141.936 to 147.709)
	rf				
	cbt				
Pentacam	et	0.369 (0.352 to 0.387)	149.026 (146.302 to 151.788)	0.363 (0.345 to 0.380)	149.174 (146.292 to 152.051)
	cbt				
	rf				
Sirius	et	0.410 (0.392 to 0.427)	143.577 (140.580 to 146.573)	0.404 (0.386 to 0.422)	144.017 (140.997 to 147.032)
	cbt				
	rf				
UBM and Pentacam	et	0.450 (0.426 to 0.475)	137.316 (134.338 to 140.143)	0.452 (0.427 to 0.477)	137.046 (134.078 to 140.092)
	cbt				
	lgb				
UBM and Sirius	cbt	0.467 (0.439 to 0.494)	132.698 (129.679 to 135.779)	0.468 (0.442 to 0.494)	132.985 (128.982 to 136.821)
	et				
	lgb				
Pentacam and Sirius	cbt	0.418 (0.402 to 0.435)	141.930 (138.869 to 145.154)	0.417 (0.400 to 0.433)	141.890 (138.807 to 144.869)
	et				
	lgb				
UBM and Pentacam and Sirius	cbt	0.504 (0.480 to 0.527)	129.893 (127.758 to 132.046)	0.499 (0.470 to 0.528)	130.655 (128.949 to 132.111)
	et				
	lgb				
Only other devices	et	0.208 (0.175 to 0.242)	175.31 (169.631 to 181.099)	0.210 (0.181 to 0.240)	172.132 (167.290 to 176.934)
	rf				
	cbt				

*cbt* CatBoost regressor; *et* Extra trees regreessor; *rf* Random forest regressor; *gbr* Gradient boosting regressor; *lgb* Light gradient boosting machine; *MAE* mean absolute error; *CI* Confidence interval

**Fig. 1:**
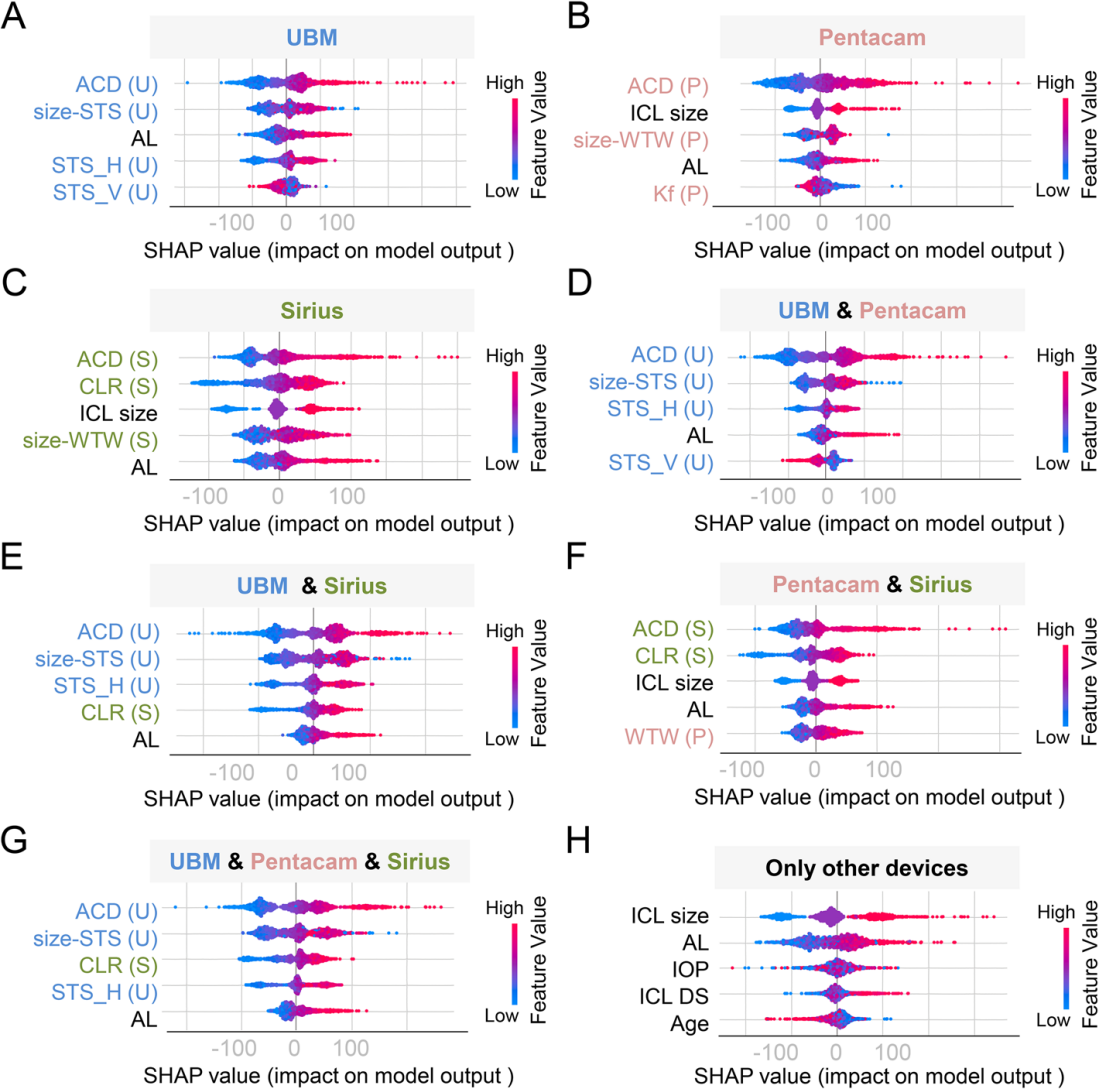
Top five features in best-performing models for vault prediction using data from different devices and combinations. SHAP summary plots for the top five features in vault prediction algorithms with data from different ophthalmic devices and combinations (**A–G**), and various devices without UBM, Pantacam, and Sirius (**H**). The higher the SHAP value for each feature, the higher risk of vault increase. *SHAP* Shapley Additive Explanations; *ACD* central anterior chamber depth; *STS_H* horizontal sulcus-to-sulcus; *STS_V* vertical sulcus-to-sulcus; *ICL* Implantable Collamer Lens; *AL* axial length; *Kf* flat keratometry; *Ks* steep keratometry; *ACV* anterior chamber volume; *size-STS* the difference between ICL size and STS_H; *WTW* horizontal white-to-white; *size-WTW* the difference between ICL size and WTW; *CLR* crystalline lens rise; *SE*: spherical equivalent; *IOP* intraocular pressure; *UCVA* uncorrected distance visual acuity

After comparing the performance of various models based on parameters from various instrument combinations, we identified the best-performing model and applied the SHAP approach for a more in-depth understanding of the model’s decision-making process. This approach enables us to uncover valuable insights into the impact of individual features and to improve the transparency and reliability of our model. Figure 1 displays the SHAP value summary chart, with the top 5 variables of each combination presented. The chart reveals the correlation between the high or low SHAP values and the prediction model. We observed that ACD consistently ranked first, and the high values of ACD appeared more on the side of the higher vault in all device combinations (Fig. 1A–G). These results indicated that ACD’s contribution to value was the largest of all features, and the SHAP values of ACD were positively correlated with the post-operative vault. Moreover, among the combinations containing UBM, size-STS, and STS showed significant contributions to the vault prediction (Fig. 1A, D, E, G); in the combinations without UBM, WTW or size-WTW played essential roles (Fig. 1B, C, F). Notably, AL consistently played an important role in all device combinations' predictive models, indicating its relevance in vault prediction (Fig. 1A–G). Furthermore, CLR, a lens-related parameter from Sirius, exhibited excellent predictive importance, even ranking third among the parameters of all devices used.

### Machine learning algorithms based on various ophthalmic device combinations perform well on optimal ICL size prediction

As listed in Table 3, most models achieved excellent optimal ICL size prediction performance. The combination containing Pentacam, Sirius, and UBM achieved the highest ACC [validation set: 0.891 (95% CI 0.879–0.904); test set: 0.895 (95% CI 0.883–0.907)] and AUC [validation set: 0.926 (95% CI 0.913–0.939); test set: 0.928 (95% CI 0.916–0.941)]. As expected, without these three devices resulted in poor predictions of whether ACC [validation set: 0.544 (95% CI 0.530–0.558); test set: 0.543 (95% CI 0.539–0.547)] or AUC [validation set: 0.636 (95% CI 0.627–0.646); test set: 0.634 (95% CI 0.630–0.638)]. The UBM-only model performs best among all single-device models [validation set: ACC = 0.834 (95% CI 0.828–0.840), AUC = 0.905 (95% CI 0.898–0.912); test set: ACC = 0.837 (95% CI 0.830–0.843), AUC = 0.906 (95% CI 0.897–0.914)], and even outperforms the combination of Pentacam and Sirius.

**Table 3:** Performance of the classification models for optimal ICL size prediction

Device (s)	Top 3 algorithms	Validation set		Test set	
		ACC (95% CI)	AUC (95% CI)	ACC (95% CI)	AUC (95% CI)
UBM	et	0.834 (0.828 to 0.840)	0.905 (0.898 to 0.912)	0.837 (0.830 to 0.843)	0.906 (0.897 to 0.914)
	rf				
	cbt				
Pentacam	et	0.682 (0.667 to 0.698)	0.803 (0.789 to 0.817)	0.682 (0.667 to 0.697)	0.802 (0.789 to 0.817)
	lgb				
	rf				
Sirius	et	0.701 (0.689 to 0.713)	0.809 (0.797 to 0.823)	0.695 (0.682 to 0.708)	0.808 (0.795 to 0.821)
	cbt				
	lgb				
UBM and Pentacam	et	0.851 (0.842 to 0.859)	0.908 (0.898 to 0.918)	0.855 (0.847 to 0.864)	0.911 (0.901 to 0.921)
	rf				
	cbt				
UBM and Sirius	lgb	0.863 (0.855 to 0.872)	0.915 (0.903 to 0.927)	0.862 (0.853 to 0.870)	0.913 (0.901 to 0.926)
	cbt				
	xbt				
Pentacam and Sirius	et	0.728 (0.714 to 0.742)	0.816 (0.812 to 0.819)	0.734 (0.720 to 0.749)	0.818 (0.814 to 0.821)
	rf				
	cbt				
UBM and Pentacam and Sirius	cbt	0.891 (0.879 to 0.904)	0.926 (0.913 to 0.939)	0.895 (0.883 to 0.907)	0.928 (0.916 to 0.941)
	rf				
	lgb				
Only other devices	et	0.544 (0.530 to 0.558)	0.636 (0.627 to 0.646)	0.543 (0.539 to 0.547)	0.634 (0.630 to 0.638)
	rf				
	cbt				

*et* Extra trees classifier; *rf* Random forest classifier; *lgb* Light gradient boosting machine; *cbt* CatBoost classifier; *xbt* Extreme gradient boosting; *ACC* accuracy; *AUC* the area under the curve; *CI* confidence interval

**Fig. 2:**
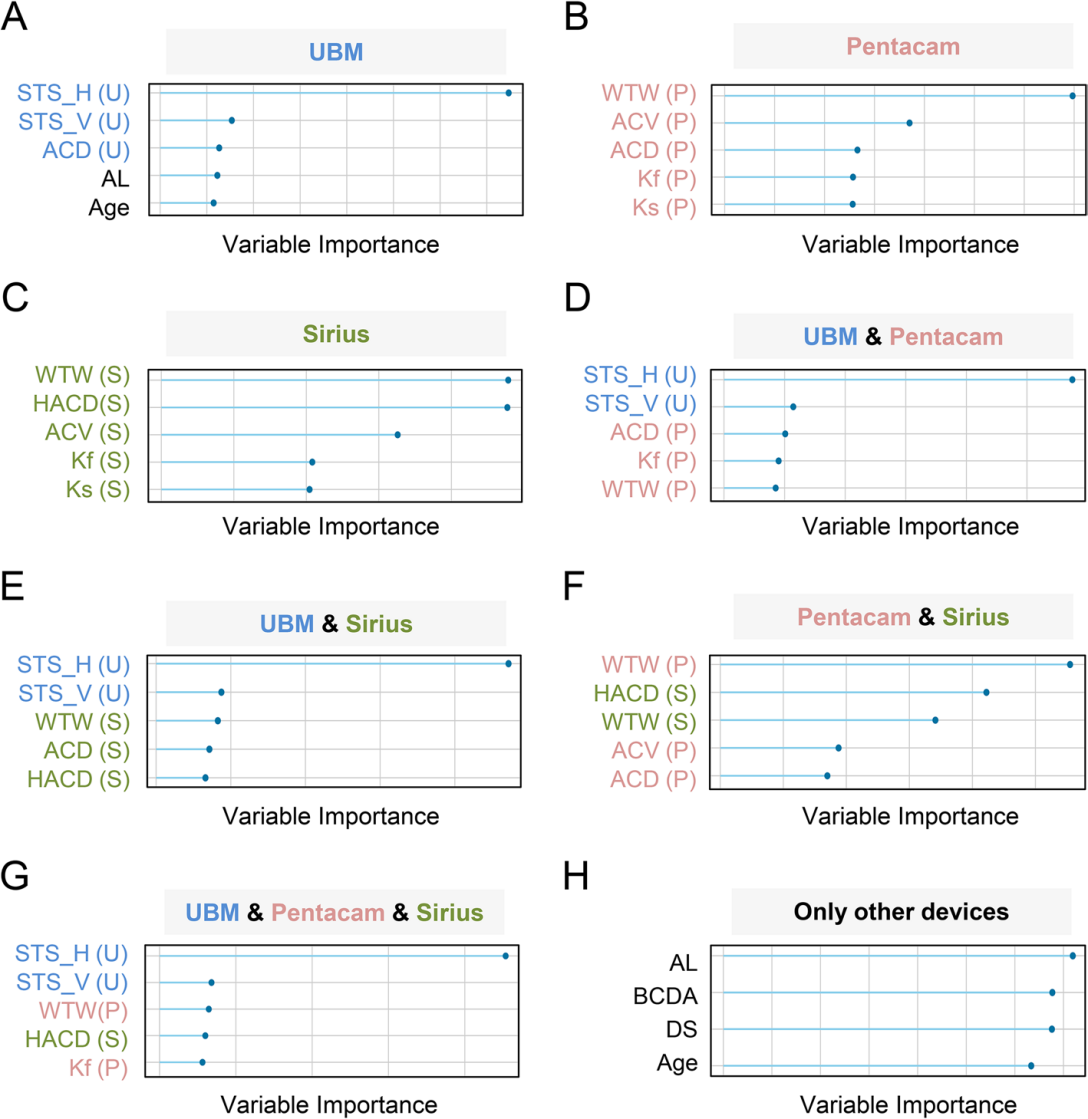
Top ranked features in best-performing models for ICL size prediction using data from different devices and combinations. Feature importance plots for the top five features in optimal ICL size prediction algorithms with data from different ophthalmic devices and combinations (**A**–**G**), and various devices without UBM, Pantacam, and Sirius (**H**). *ICL* Implantable Collamer Lens; *STS_H* Horizontal sulcus-to-sulcus; *STS_V* Vertical sulcus-to-sulcus; *ACD* Central anterior chamber depth; *AL* Axial length; *IOP* Intraocular pressure; *WTW* Horizontal white-to-white; *ACV* Anterior chamber volume; *Kf* Flat keratometry; *Ks* Steep keratometry; *HACD* Horizontal anterior chamber diameter; *DS* Spherical refraction; *DC* Cylinder refraction

The weights of features of the classification models for ICL size prediction are shown in Fig. 2. Among the combinations containing UBM, STS_H and STS_V were the two most crucial features (Fig. 2A, D, E, G); and in the combinations without UBM, WTW played an important role in prediction (Fig. 2B, C, F). Moreover, HACD made a significant contribution to ICL size prediction in the instrument combinations containing Sirius (Fig. 2C, E–G).

### Development of clinician-friendly software to facilitate the effectiveness and safety of ICL surgery

To maximize the clinical potential of our machine learning algorithms for post-operative vault and optimal ICL size prediction, we created user-friendly software specifically tailored for clinicians, without requiring an in-depth understanding of the AI algorithms. This software is designed to seamlessly integrate the predictive models into the clinical workflow, providing a valuable tool for ICL surgery planning.

**Fig. 3:**
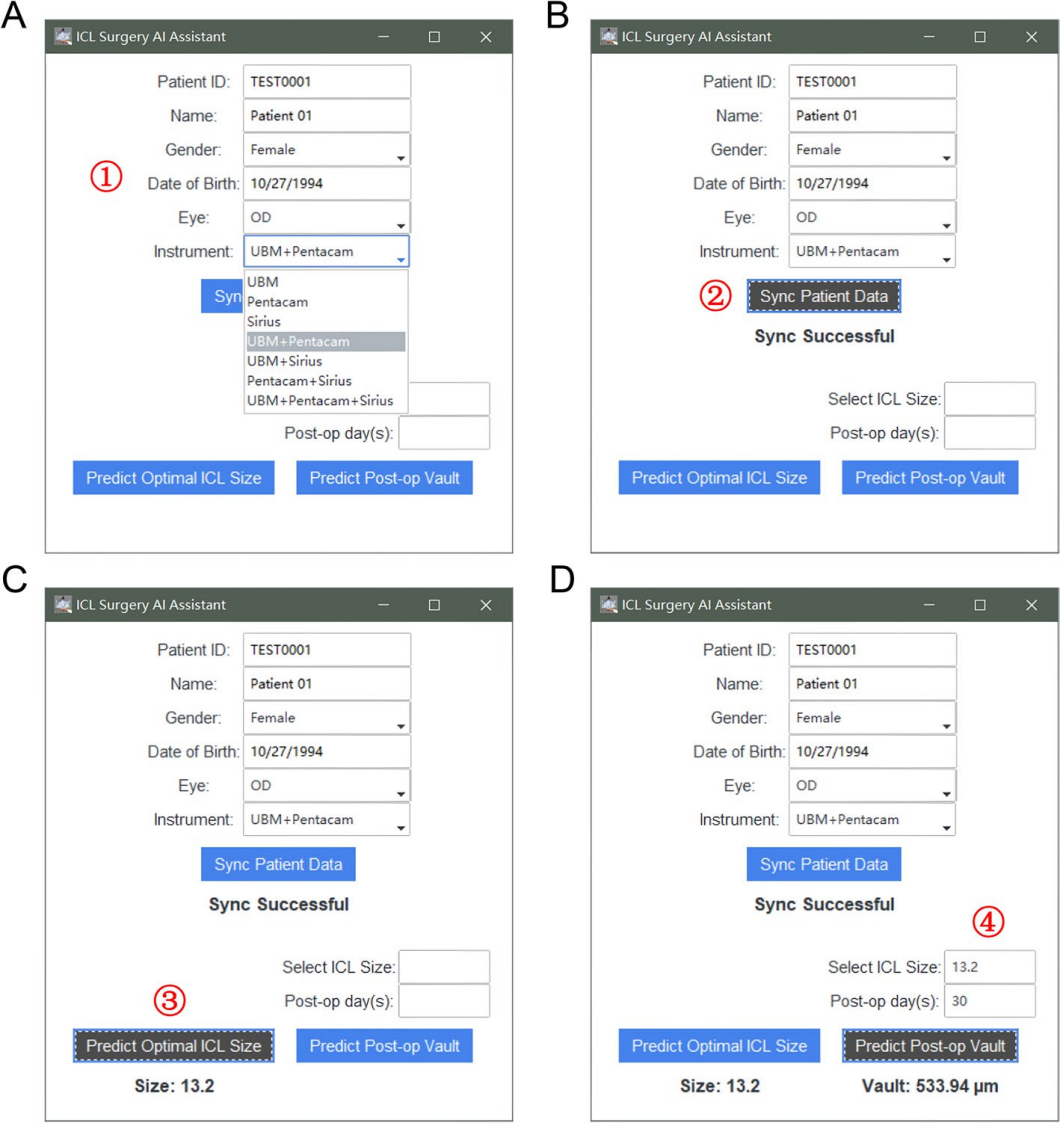
Software interface presentation of ideal ICL selection and vault prediction. **A** Input fields for patient information and device selection. **B** “Sync Patient Data” button, retrieves the necessary data for prediction based on the input patient information and selected devices. **C** “Predict Optimal ICL Size” button, generates the recommended ICL size for the patient’s eye based on the algorithm's prediction. **D** “Predict Post-operative Vault” button, calculates the expected post-operative vault at a specific timepoint after entering the planned ICL size and post-operative days

The graphical user interface is designed with clinicians in mind, offering a streamlined and intuitive layout for easy data input and navigation (Fig. 3). After opening the software, clinicians can input patient information, such as patient ID, name, gender, date of birth, eye, and the ophthalmic devices to be used in the prediction process. By clicking the “Sync Patient Data” button, the software retrieves the required data for prediction. Clinicians can then click the “Predict Optimal ICL Size” button to obtain the recommended ICL size. In addition, users can input the planned ICL size and post-operative days to predict the post-operative vault at specific timepoints.

This software would amplify the real-world applicability of our algorithms. Healthcare professionals could make more informed decisions by incorporating various AI predictive models into their decision-making process, ultimately improving patient outcomes.

## Discussion

The current study utilized the comparison of multiple algorithms, stacking ensemble learning, and data from different ophthalmic devices and combinations to predict post-operative vault and appropriate ICL size. Compared to other previous studies, our strategies exhibited improved predictive effects in both the validation sets (stratified tenfold cross-validation) and the test sets, and our models showed good interpretability.

Our study achieved precise post-operative vault prediction based on features from three optional ophthalmic devices. ACD was the most significant factor influencing the post-operative vault (Fig. 1), and previously published results support these findings, suggesting that myopic eyes with greater pre-operative ACD are predisposed to higher post-operative vaulting [20, 21]. Our findings emphasized the significance of AL in predicting post-operative vault, as it consistently contributed to the prediction models across all device combinations (Fig. 1). Therefore, AL should be taken into account when developing vault prediction models. Our work also confirmed that size-STS, ICL size, and size-WTW made essential contributions to vault prediction. Lee et al*.* found that the contribution of size-STS to the outcomes surpasses that of ICL size [22], which was basically consistent with our results. When using only single-device, we found that using the parameters of Sirius alone has the most accurate vault prediction, not UBM. We speculate that we only obtained limited parameters from the UBM for the accuracy of measurement precision, such as ACD, horizontal and vertical STS, and iris cyst-related metrics, which implied a lack of parameters related to pupil and cornea. Moreover, to improve the accuracy of prediction and the objectivity of measurement, we automatically acquired lens-related parameter CLR from Sirius rather than subjectively identified and manually measured CLR from UBM. Many studies have also elucidated that pupil size and movement [23, 24], corneal keratometry and thickness [19, 25], and CLR [21, 26] were critical in vault prediction. Therefore, due to different mechanisms regulating the vault, Sirius might be more accurate in predicting post-operative vault, because it provided more comprehensive parameters than UBM in the current study. There are reasons to believe that acquiring more parameters from UBM or directly utilizing image data for model training might further improve the accuracy of vault prediction. Noteworthy, Pentacam was also worse than Sirius in predicting vault. One plausible explanation is that Pentacam lacks CLR, which indirectly reflects the importance of lens-related factors in post-operative vault prediction. For a combination of two ophthalmic devices, our results showed that UBM combined with Sirius has the best predictive ability. These results deduce that the parameters from both UBM and Sirius could fit the shape of the anterior chamber and the posterior chamber to a large extent; thus, this combination improves the predictive accuracy.

In our study, most device combinations established models with relatively good prediction accuracy for determining optimal ICL size (Fig. 2), which was higher than previous studies. We found that horizontal STS was always the most crucial factor when using data from a single ophthalmic device or multiple devices to select ICL size, as long as the input data source contains UBM (Fig. 3A, D, E G). Many previous studies supported these results. For example, Reinstein et al*.*[7] and Wachler et al*.*[19] reported that the horizontal STS by UBM could provide excellent vault predictability for selecting the optimal ICL size, proving our results' correctness. It is also noteworthy that the vertical STS ranked second to the horizontal STS in selecting the appropriate ICL size. Since our study removed the effect of multicollinearity, the probable explanation of the above results could be that horizontal and vertical STSs would jointly affect the rotational stability of the implanted ICL, rather than vertical STSs being highly correlated with horizontal ones. Lin et al*.* indicated that the physiologic nystagmus of the eyeballs is likely to cause ICL rotation toward the direction with the larger diameter [27], and thus the larger the vertical STS might cause a greater likelihood of rotating the horizontally placed ICL and the greater the vault change. Therefore, ophthalmologists should consider the size of the placed ICL with reference to the STSs in different directions. If only Scheimpflug tomographers are used (Pentacam, Sirius, or combination without UBM), WTW is the essential parameter determining ICL size selection (Fig. 2B, C, F) which is in line with clinical experience. Given that STS was in the top five significant contributions to whether post-operative vault or optimal ICL size prediction and was always better than WTW, and excellent ICL selection prediction could be achieved with only the parameters from UBM (Table 3), there is a need for greater attention to UBM in the perioperative period of ICL surgery.

In addition, our results demonstrated that in the options of using Sirius' data (Fig. 2C, E–G), HACD contributed to the feature importance in ICL size prediction, indicating this feature could not be neglected in clinical practice. HACD is defined as the distance between the vertices of iridocorneal angles on the horizontal Scheimpflug image [28], and it is also referred to as angle-to-angle distance (ATA). Many studies confirmed that ATA correlates strongly with appropriate ICL size [10, 26], which partially explains why HACD/ATA was a critical feature in the ICL size selection models.

Despite its strengths, we also acknowledge some limitations. First, our study was based on a retrospective data analysis and should be confirmed through further prospective studies. Second, our proposed models were based on our single-center data set. We anticipate that larger, more diverse ICL cases with multi-center studies should be performed to assess the feasibility of our method. In future studies, we will try to overcome the above limitations and improve prediction accuracy and generality.

## Conclusions

In summary, applying the multiple supervised ML approaches to multi-modal data effectively predicted post-operative vault and ICL sizing. More importantly, considering equipment conditions differ among hospitals, we developed different ML models to suit different devices and device combinations. This study could help ophthalmologists in various hospitals and clinics predict vault precisely, thus enabling appropriate selection of the ICL size and improving the safety of ICL implantation.

## Materials and methods

### Patients

Patients who underwent routine pre-operative examinations for V4c ICL (EVO ICL, STAAR Surgical) surgery between September 2020 and September 2022 were included in this cross-sectional study. The essential inclusion criteria were as follows: no preexisting ocular pathology other than refractive error, no previous ocular surgery or trauma, and endothelial cell density greater than or equal to 2000/mm^2^. Finally, 1941 eyes of 1941 patients (mean age: 26.38 ± 5.07 years) were included in this study.

### Measurements

Pre-operative routine examinations were conducted as follows: (1) uncorrected (UDVA) and corrected distance visual acuity (CDVA), spherical refraction (DS), cylinder refraction (DC), manifest and cycloplegic refraction, intraocular pressure (IOP), axial length (AL) measurement (IOLmaster 500, Carl Zeiss, Germany) were completed; (2) slit-lamp examination, fundus examination, endothelial cell density (CEM-530, NIDEK, Japan) were completed; (3) Pentacam HR (Oculus Optikerate, Carl Zeiss, Germany) was used for measuring horizontal white-to-white (WTW), central corneal thickness (CCT), central anterior chamber depth (ACD), corneal volume (CV), anterior chamber volume (ACV), anterior chamber angle (ACA), flat keratometry (Kf), and steep keratometry (Ks); (4) Sirius (Costruzione Strumenti Oftalmici, Italy) was used for measuring WTW, CCT, CV, ACV, ACA, ACD, Kf, Ks, photopic/mesopic/scotopic pupil diameter (PD), symmetry index front and back (SIf, SIb), keratoconus vertex front and back (KVf, KVb), Baiocchi-Calossi-Versaci index front and back (BCVf, BCVb), horizontal anterior chamber diameter (HACD), and crystalline lens rise (CLR); (5) ACD, horizontal STS diameter (STS_H), vertical STS diameter (STS_V), and the number of iridociliary cysts were measured using ultrasound bio-microscopy (UBM) (SW-3200L, Suowei, China) by the same experienced technician. Three repeated measurements were made to ensure the quality of the image, and each scan should display the strongest reflection of the cornea, anterior and posterior capsule of the lens, and the widest STS distance. The radial scans were captured when zonular fibers and the longest ciliary process were presented simultaneously; (6) information on ICL, including DS of ICL, DC of ICL, type of ICL, the difference between ICL size and horizontal WTW (size-WTW) or horizontal STS (size-STS) were included for vault prediction; and (7) the objective vault was measured using AS-OCT (RTVue XR, Optovue, the United States).

### Surgical procedure

The choice of ICL size was determined through a comprehensive assessment by three experts, all of whom were board-certified and had an average of 5 years of experience in performing ICL surgery.

The ICL implants were all carried out by the same skilled surgeon, Dr. Yu KM. Prior to the procedure, the pupils were dilated using Mydrin-P (a combination of 0.5% tropicamide and 0.5% phenylephrine) from Santen in Osaka, Japan. The V4c ICL was inserted through a 3.0 mm temporal clear corneal incision with the aid of an injector cartridge from STAAR Surgical Co. in Monrovia, CA, USA. After filling the anterior chamber with viscoelastic, the ICL was positioned in the posterior chamber and aligned to the desired cylinder axis using a modified manipulator. The remaining viscoelastic was then removed and replaced with the balanced salt solution. Subsequently, an antibiotic solution of either Cefuroxime sodium or Vancomycin was injected into the anterior chamber.

### Data preprocessing

All the samples did not contain missing values. The data were randomly divided into the training set and test set according to the ratio of 8:2. All programs involving the model training and testing stages were executed on a workstation with a 32-core NVIDIA Tesla V100S GPU and 2 T GB of RAM. We set up the environment with a train-test split of 0.8 and GPU usage. Data preprocessing techniques, such as removing outliers, multicollinearity, and feature selection with a threshold of 0.8, were applied. More specifically, outliers were identified through PCA linear dimensionality reduction using the Singular Value Decomposition technique, and 0.025 of the values on each side of the distribution’s tail were dropped from training data; A feature was considered a low variance feature and removed from the data set if it met two crucial conditions: (count of unique values in a feature)/(sample size) < 10%, and (count of most common value)/(count of second most common value) > 20 times; When two features were highly correlated with each other (threshold ≥ 0.9), the feature that was less correlated with the target variable was dropped; Feature selection (working with selected features instead of all the features) reduces the risk of over-fitting, improves accuracy, and decreases the training time [29]; A subset of features was selected using various permutation importance techniques, including Random Forest, Adaboost, and Linear correlation with the target variable. All measurements were repeated three times by skilled physicians, and the average value was taken.

### Model development

**Fig. 4:**
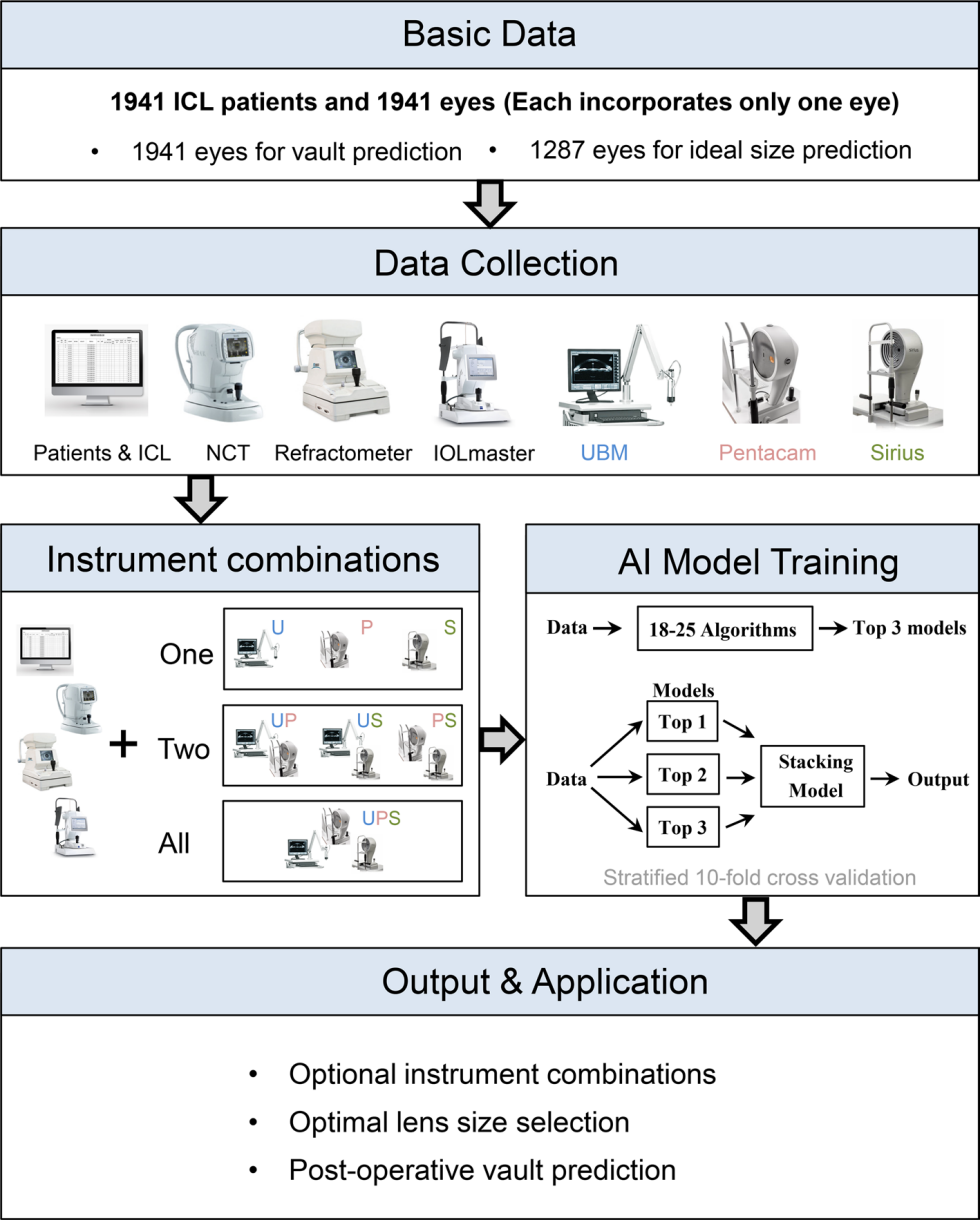
Overall study pipeline. The workflow of predicting post-operative vault ideal ICL size using multiple artificial intelligence algorithms, stacking ensemble learning, and data from various ophthalmic devices and combinations

The research procedure is illustrated in Fig. 4. We compared 25 supervised regression algorithms (for vault prediction, Additional file 1: Table S1) and 18 supervised classification models (for ICL size selection, Additional file 1: Table S1) by the PyCaret library, a python-based framework for automating machine learning workflows [30]. Stratified tenfold cross-validation was used for metric evaluation on the training set. The top 3-performing models were selected for further development. For each of the top three models, we used the tune_model() function in PyCaret to optimize their hyperparameters through a random grid search within a predefined search space. The ensemble technique, known for improving models by combining several models, was utilized to enhance the model's accuracy and reduce prediction variability. Consequently, we adopted a stacking ensemble strategy [31]. In this strategy, the predictions of the three optimized models from 9 out of 10 validations were used as input. Logistic regression was applied as the meta-model in the second layer for classification experiments, while linear regression was used for regression experiments.

### Model evaluation

To quantitatively evaluate the prediction performance of the regression models for post-operative vault prediction, we used two evaluation metrics, mean absolute error (MAE, the average absolute difference between the predicted values and the true values) and R^2^-score (the proportion of variance in the dependent variable that is explained by the independent variables in the model). For the classification models of optimal ICL size prediction, we adopted two metrics to assess the performance of the stacking ensemble models: accuracy (ACC, the number of correct predictions divided by the total number of predictions made) and the area under the curve (AUC, a performance metric for binary classification problems that summarizes the model's ability to distinguish between positive and negative classes). A high score of AUC indicates that the model has high quality in differentiating its classes.

### Model interpretation

In light of the importance of model interpretability, we employed the SHAP (Shapley Additive Explanations) method, a game-theoretic technique for explaining the output of machine learning models [32]. SHAP values provide quantified contributions, intuitively demonstrating the effect of each feature on shifting the model output from the base value [33, 34].

## Supplementary Information

**Additional file 1: Table S1.** Algorithms used for model development.

## Data Availability

The data sets used and analyzed during the current study are available from the corresponding authors upon reasonable request.

## Abbreviations

ICL: Implantable collamer lens
AI: Artificial intelligence
CI: Confidence interval
MAE: Mean absolute error
AUC: Area under the curve
ML: Machine learning
AS-OCT: Anterior segment optical coherence tomography
UBM: Ultrasound bio-microscopy
STS: Sulcus-to-sulcus
STS_H: Horizontal STS diameter
STS_V: Vertical STS diameter
WTW: White-to-white
UDVA: Uncorrected distance visual acuity
CDVA: Corrected distance visual acuity
DS: Spherical refraction
DC: Cylinder refraction
IOP: Intraocular pressure
AL: Axial length
CCT: Central corneal thickness
ACD: Anterior chamber depth
CV: Corneal volume
ACV: Anterior chamber volume
ACA: Anterior chamber angle
Kf: Flat keratometry
Ks: Steep keratometry
PD: Pupil diameter
HACD: Horizontal anterior chamber diameter
CLR: Crystalline lens rise
