# Supplementary material for: Predicting post-operative vault and optimal implantable collamer lens size using machine learning based on various ophthalmic device combinations
Source: Biomed Eng Online. 2023 Jun 15;22:59. doi: 10.1186/s12938-023-01123-w (PMC10268449; doi:10.1186/s12938-023-01123-w)
Supplement: Supplementary file 1 — Additional file 1: Table S1. Algorithms used for model development. [file 12938_2023_1123_MOESM1_ESM.docx]

**Additional Table S1. Algorithms used for model development.**

| **ID** | **Regression（Vault Prediction）** | **ID** | **Classification（Size Selection）** |
| --- | --- | --- | --- |
| 1 | Linear Regression | 1 | Logistic Regression |
| 2 | Lasso Regression | 2 | K Neighbors Classifier |
| 3 | Ridge Regression | 3 | Naive Bayes |
| 4 | Elastic Net | 4 | Decision Tree Classifier |
| 5 | Least Angle Regression | 5 | SVM - Linear Kernel |
| 6 | Lasso Least Angle Regression | 6 | SVM - Radial Kernel |
| 7 | Orthogonal Matching Pursuit | 7 | Gaussian Process Classifier |
| 8 | Bayesian Ridge | 8 | MLP Classifier |
| 9 | Automatic Relevance Determination | 9 | Ridge Classifier |
| 10 | Passive Aggressive Regressor | 10 | Random Forest Classifier |
| 11 | Random Sample Consensus | 11 | Quadratic Discriminant Analysis |
| 12 | TheilSen Regressor | 12 | Ada Boost Classifier |
| 13 | Huber Regressor | 13 | Gradient Boosting Classifier |
| 14 | Kernel Ridge | 14 | Linear Discriminant Analysis |
| 15 | Support Vector Regression | 15 | Extra Trees Classifier |
| 16 | K Neighbors Regressor | 16 | Extreme Gradient Boosting |
| 17 | Decision Tree Regressor | 17 | Light Gradient Boosting Machine |
| 18 | Random Forest Regressor | 18 | CatBoost Classifier |
| 19 | Extra Trees Regressor |  |  |
| 20 | AdaBoost Regressor |  |  |
| 21 | Gradient Boosting Regressor |  |  |
| 22 | MLP Regressor |  |  |
| 23 | Extreme Gradient Boosting |  |  |
| 24 | Light Gradient Boosting Machine |  |  |
| 25 | CatBoost Regressor |  |  |
